# Co-occurrence of Vogt–Koyanagi–Harada disease, diabetic retinopathy, and advanced chronic kidney disease: a case report

**DOI:** 10.3389/fmed.2026.1733153

**Published:** 2026-01-20

**Authors:** Yutong Huang, Yifei Wang, Qian Ren

**Affiliations:** 1 Department of Ophthalmology, Shijiazhuang People's Hospital, Shijiazhuang, Hebei, China; 2 Graduate School, Hebei Medical University, Shijiazhuang, Hebei, China

**Keywords:** adalimumab, chronic kidney disease, diabetic retinopathy, serous retinal detachment, Vogt–Koyanagi–Harada disease

## Abstract

**Objective:**

This study aimed to report a case of Vogt–Koyanagi–Harada (VKH) disease in a 53-year-old man with renal failure and diabetes, highlighting the challenges in diagnosing VKH amidst complex systemic comorbidities.

**Methods:**

A case report was conducted through a retrospective review of the patient’s medical history, clinical presentations, diagnostic workup, and treatment course.

**Results:**

A 53-year-old man with renal failure, hypoalbuminemia, and diabetes presented with blurred vision. Initial findings included bilateral serous retinal detachment with subretinal septa, choroidal thickening, and left eye vitreous hemorrhage, without anterior chamber inflammation. Subsequent anterior segment inflammation and a positive response to combined corticosteroids and adalimumab confirmed VKH.

**Conclusion:**

This case underscores the diagnostic complexity of VKH in patients with multiple systemic conditions. Corticosteroid management in such individuals requires meticulous attention, emphasizing the need for close monitoring of blood glucose and overall systemic status during treatment.

## Background

Bilateral serous retinal detachment with choroidal thickening is a common fundus manifestation of Vogt–Koyanagi–Harada (VKH) disease, among which cases complicated with diabetic retinopathy and renal failure are rare, increasing the difficulty of disease diagnosis. Immunopathological studies have established that Vogt–Koyanagi–Harada (VKH) disease is a CD4 + T cell-mediated autoimmune disorder directed against melanocytes (1). In Vogt–Koyanagi–Harada (VKH) syndrome, T cells mediate autoimmunity through the recognition of tyrosinase-derived peptides, implicating tyrosinase as a central autoantigen (2). However, clinical practice shows that not all patients fit these typical manifestations, and the diversity and atypicality of clinical presentations often pose challenges to early diagnosis.

## Case presentation

**Figure 1:**
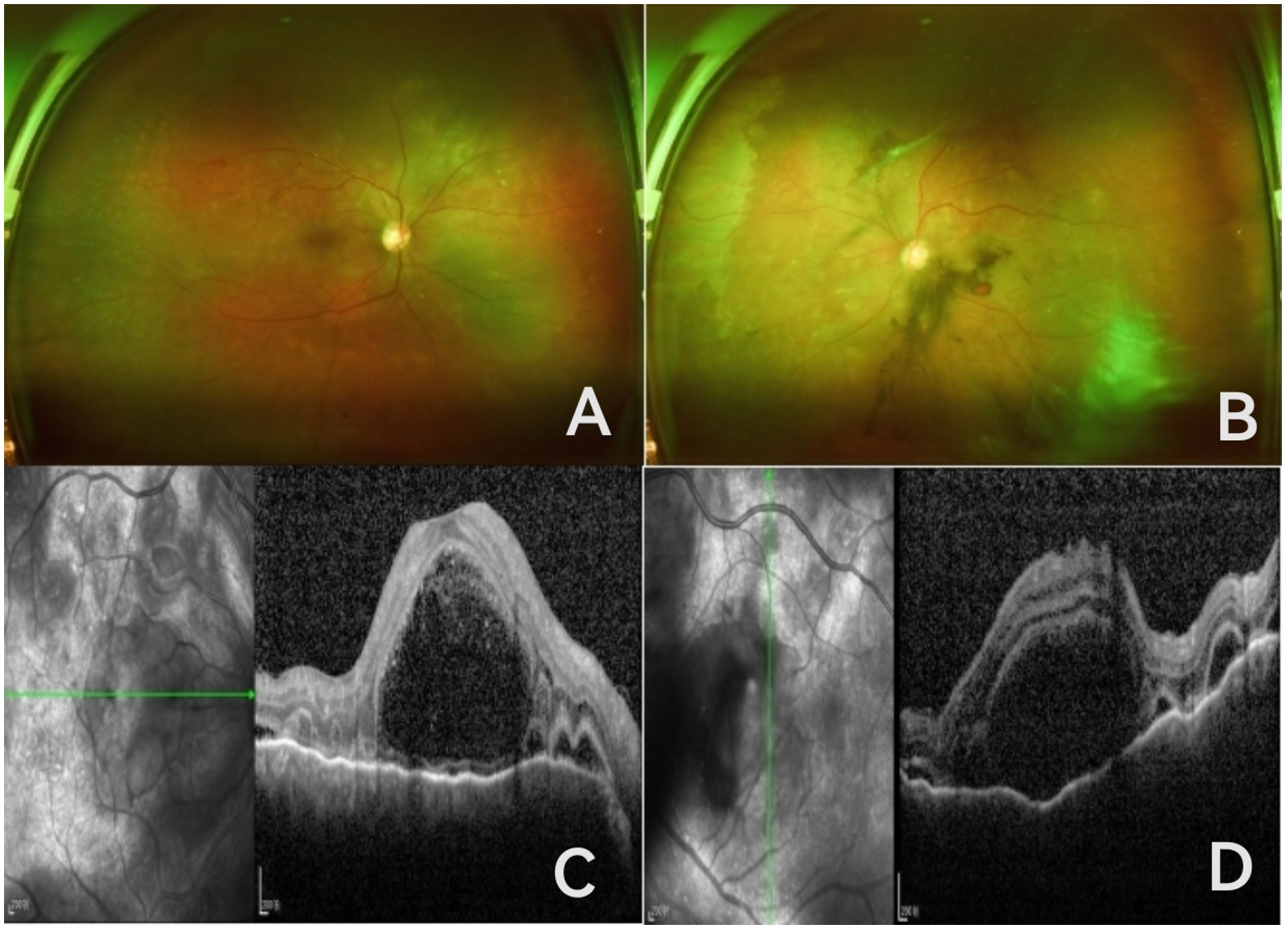
Ultra-widefield fundus images of both eyes show microaneurysms, punctate and patchy hemorrhages, and yellow-white lesions on the retinas of both eyes; the left eye has vitreous hemorrhage, with the optic disc and retinal neovascularization barely visible; **(A,B)** OCT of both eyes reveals multiseptated subretinal fluid accumulation accompanied by subretinal septa and choroidal thickening **(C,D)**.

A 53-year-old man was hospitalized in the nephrology department for treatment of renal failure accompanied by vision loss in both eyes for 1 week. Before this episode, the patient had no symptoms such as cold, fever, tinnitus, vomiting, or graying of hair, only headache, localized horizontally above both eyes to the top of the skull, described as stabbing pain. Fundus photography showed microaneurysms, dot-and-blot hemorrhages, and exudates in both eyes, with vitreous hemorrhage in the left eye. OCT showed multiloculated subretinal fluid with subretinal septa and choroidal thickening in both eyes (Figure 1). The patient had a 10-year history of type 2 diabetes and a 3-year history of stage 4 chronic kidney disease. He was on insulin for glycemic control and had no history of ocular surgery.

Physical examination: BP 138/81 mmHg, BCVA was hand motion in both eyes, intraocular pressure right eye 18 mmHg, left eye 19 mmHg, transparent corneas in both eyes, clear anterior chambers, no anterior chamber cells, cortical lens opacities, vitreous opacity in the right eye, vitreous hemorrhage in the left eye; fundus of the right eye showed retinal arteriovenous crossing compression, visible microaneurysms, punctate and patchy hemorrhages and exudates; the left eye showed indistinct retinal and optic disc neovascularization and scattered yellow-white punctate lesions. Given the severe subretinal fluid (SRF) but lack of anterior chamber inflammation, the differential diagnosis considers VKH syndrome, hypoproteinemia, or exudative retinal detachment related to renal failure, or complications of diabetic retinopathy. Three days later, several keratic precipitates of varying sizes were visible on the posterior corneal surface in both eyes, anterior chamber cell activity (3+), and a Koeppe nodule was observed at the 12 o’clock position of the left iris. Combining the typical signs of granulomatous anterior uveitis, multicystic SRF, choroidal thickening, and newly developed anterior segment inflammation, the clinical diagnosis is VKH syndrome. OCT from 2 years ago showed punctate hyperreflective signals within the retinal layers in both eyes; OCTA showed microaneurysms and small areas of non-perfusion in both eyes with incompletely formed perifoveal capillary arcades. Due to the patient’s poor systemic condition, FFA and ICGA examinations were not performed. Tests for HIV, syphilis, hepatitis B, and hepatitis C antibodies were all negative. Hematological tests showed white blood cell count 9.67 × 10^9^/L, red blood cell count 3.75 × 10^12^/L, hemoglobin 108 g/L, pro-BNP 3091 pg./mL, HbA1c 8.9%, total glycosylated hemoglobin 10.4%, fasting blood glucose 10.0 mmol/L, elevated D-dimer quantitative 247.17 ng/mL, elevated urea 16.0 mmol/L, elevated creatinine 274 μmol/L, decreased albumin 34.7 g/L, estimated glomerular filtration rate (eGFR) 17.73, decreased transferrin 1.59 g/L, transferrin saturation 17.12%, serum iron 8.20 μmol/L; unsaturated iron-binding capacity, total iron-binding capacity, complement C3 and C4, immunoglobulins A, G, M, ferritin, and parathyroid hormone were within normal ranges. Carotid Doppler ultrasound showed bilateral carotid atherosclerosis with plaque formation on the left side. Cranial CT showed bilateral basal ganglia lacunar infarcts.

**Figure 2:**
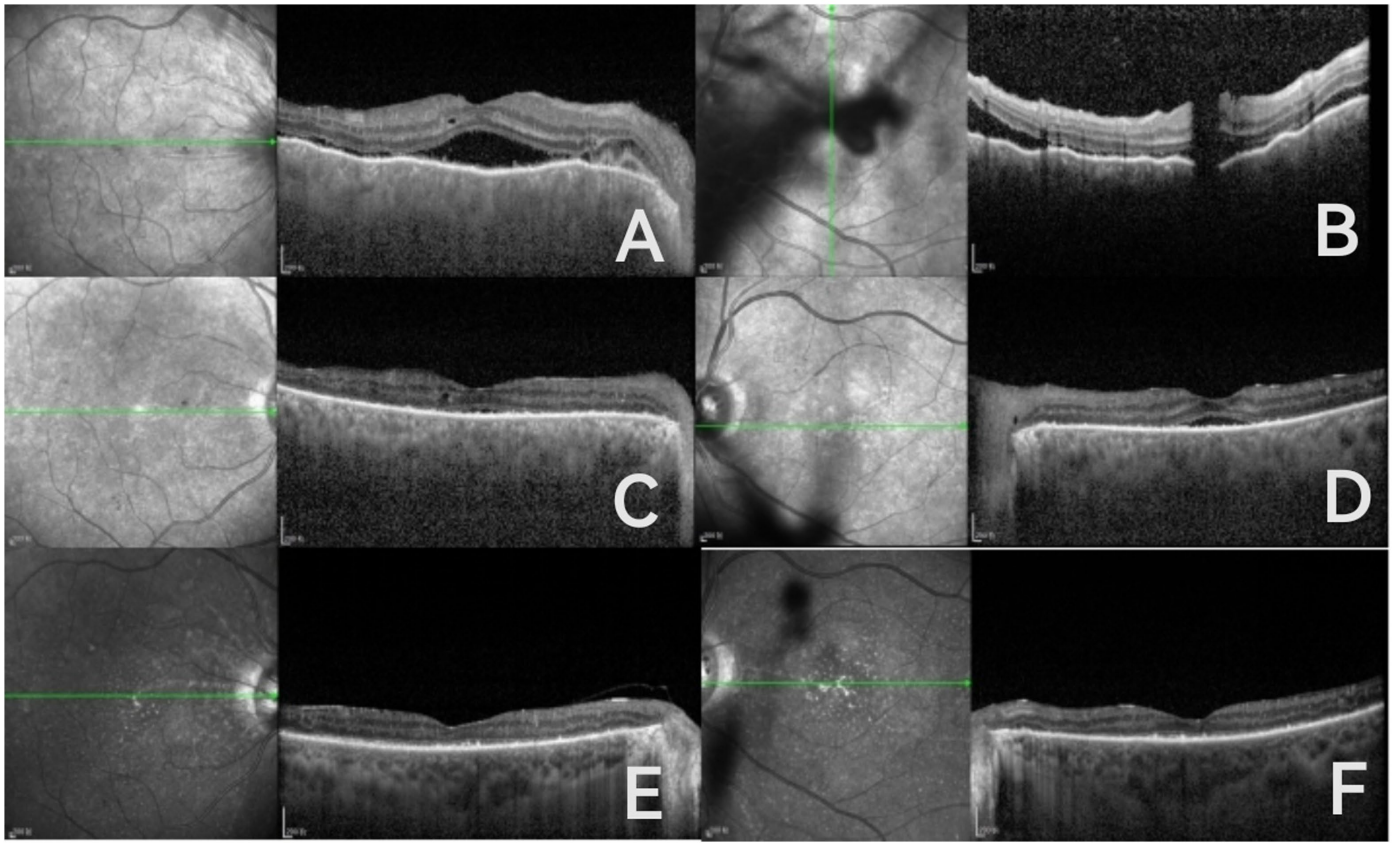
One week later: resolution of subretinal fluid and retinal flattening in both eyes; persistent choroidal folds (both eyes); and remaining vitreous hemorrhage in the left eye **(A,B)**. One month later: OCT of both eyes showed residual subretinal fluid in both eyes, punctate hyperreflective signals between retinal layers, and a blurred ellipsoid zone **(C,D)**. Four months later: OCT shows a flat retina, subretinal fluid absorption, and a blurred ellipsoid zone **(E,F)**.

The patient was diagnosed with VKH and given methylprednisolone. On the third day of treatment, the patient developed tinnitus accompanied by hearing loss. The patient’s overall condition was poor, and his blood glucose control was unsatisfactory, so the treatment was changed to oral methylprednisolone tablets combined with subcutaneous injection of adalimumab. One week later, the patient’s best-corrected visual acuity (BCVA) was 20/1000, with optical coherence tomography (OCT) showing partial resolution of subretinal fluid and a flattened retina compared to previous findings (Figure 2). One month later, the patient’s BCVA was 0.04 in both eyes. Ophthalmic examination showed that the anterior chamber inflammatory reaction in both eyes had subsided, multifocal serous retinal detachment in the posterior pole was flatter than before, patchy retinal hemorrhages and exudates were visible, and macular edema was reduced compared to before. OCT of both eyes still showed a small amount of subretinal fluid and punctate hyperreflective signals between layers, with blurring of the ellipsoid zone (Figure 2).

After administering methylprednisolone, the patient’s fasting blood glucose is 12 mmol/L. Please consult the endocrinology department, and subsequently add sitagliptin and liraglutide for glycemic control. After 4 months, the patient reports good blood glucose control, with a bilateral best-corrected visual acuity (BCVA) of 0.1, clear cornea, no anterior chamber flare, round pupils, and light reflex present. There are scattered punctate hemorrhages and exudates in both fundi, with multifocal serous retinal detachment compared to before, and the retina is flat. OCT shows a flat retina, subretinal fluid absorption, and a blurred ellipsoid zone (Figure 2). Due to the complex systemic condition of the patient, long-term follow-up is still ongoing. Currently, the combination treatment effectively controls ocular inflammation while maintaining good glucose control with the use of hypoglycemic medications.

## Discussion

Vogt–Koyanagi–Harada (VKH) disease is a systemic autoimmune uveitis characterized by bilateral diffuse choroiditis, clinically commonly presenting with anterior segment inflammation, serous retinal detachment, choroidal edema and thickening, as well as systemic manifestations related to central nervous system involvement (headache, cerebrospinal fluid lymphocytosis), auditory symptoms (tinnitus, hearing loss), and skin and hair involvement (vitiligo, poliosis). The simultaneous occurrence of VKH, diabetic retinopathy, and chronic kidney disease is relatively uncommon in clinical practice. As reported in the literature, serous retinal detachment can occur secondary to renal failure or systemic conditions such as SLE, often due to factors such as hypertension, fluid overload, or hypoalbuminemia (3–7). Vitreous fluid flows through the retina and retinal pigment epithelium, which is an important mechanism for maintaining adhesion between the two (8). This fluid movement is jointly regulated by the colloid osmotic pressure of the choroid, the ion pumps of the retinal pigment epithelium cells, and intraocular pressure (9, 10). Serum albumin serves as a major factor in the colloid osmotic pressure of the circulatory system; its level drop reduces choroidal osmotic pressure, leading to the accumulation of subretinal fluid, resulting in serous retinal detachment (8, 11, 12). Exudative retinal lesions associated with hypoproteinemia may be closely related to changes in choroidal osmotic pressure and the immune environment. The patient in this case presents the following features: ① SRF shows multicystic subretinal compartments, which are typical in VKH and rare in simple hypoproteinemia-related SRF; ② Accompanied by choroidal thickening, suggesting inflammatory factors; ③ Most crucially, with anti-inflammatory treatment, SRF absorption occurs, and the anterior segment inflammatory response subsides. Although this patient has hypoalbuminemia and renal failure—factors that could lead to serous detachment—the subsequent appearance of anterior segment inflammation and positive response to steroid treatment are vital for the diagnosis of VKH.

Vogt–Koyanagi–Harada (VKH) syndrome is characteristically presented as early pinpoint hyperfluorescent spots and late multifocal lake-like fluorescence accumulations in fluorescein angiography (FFA); indocyanine green angiography (ICGA) typically shows choroidal hypofluorescence or disc hyperfluorescence. This case study was limited by the patient’s systemic condition, which prevented the completion of FFA and ICGA examinations. However, combining its anterior segment findings with optical coherence tomography (OCT) images allows for a clear diagnosis of VKH syndrome. Although ICGA is considered the gold standard for assessing choroidal status, its invasive nature and potential dye-related complications limit its application in patients with poor systemic conditions. Furthermore, OCT and optical coherence tomography angiography (OCTA) help dynamically assess the disease activity, recurrence, and prognosis of VKH (13, 14). Notably, discontinuity in the outer retinal IS/OS structure and persistent macular edema are significant markers of poor visual prognosis (15). OCTA studies further reveal that the choroidal capillary density (CCVD) significantly declines during the acute uveitis phase of VKH, rises after inflammation resolution, and drops again during relapses, indicating that choroidal circulation fluctuates with inflammatory activity (16). For patients with severe renal disease and other systemic conditions who cannot tolerate fundus angiography, EDI-OCT and OCTA serve as important non-invasive, rapid imaging tools that provide effective support for disease monitoring and prognosis evaluation.

Following inflammation control, the patient’s poor vision was attributed to residual subretinal fluid, retinal photoreceptor damage, and vitreous hemorrhage. Due to poorly controlled diabetes, renal failure, and the nephrotoxicity of conventional immunosuppressants—coupled with the patient’s refusal—adalimumab was added to the regimen, leading to ocular improvement. Concurrent systemic medications included enalapril, furosemide, compound *α*-ketoacid, and sacubitril/valsartan for comprehensive management.

TNF-α is a pro-inflammatory cytokine secreted by monocytes, CD4 + T cells, mast cells, neutrophils, and natural killer cells (17). It activates various intracellular signaling pathways by binding to TNF receptors, promoting inflammatory responses (18). There are two subtypes of TNF receptors, TNF-R1 and TNF-R2; TNF-R1 mediates cytotoxicity and promotes cell proliferation and survival through related signaling pathways. TNF-*α* inhibitors exert their anti-inflammatory effects by binding to TNF receptors, blocking the interaction between TNF-α and its receptors (19, 20). Adalimumab (ADA) is a monoclonal antibody that targets tumor necrosis factor-alpha (TNF-α), thereby treating VKH disease by inhibiting this pivotal cytokine that drives T-cell activation and disrupts the blood–retinal barrier. It has shown efficacy in controlling symptoms, improving vision, preventing relapses, and maintaining good safety profiles. It effectively relieves refractory VKH and reduces the dependence on corticosteroids for newly onset VKH (21–27). In the background of renal failure and diabetes, a persistent micro-inflammatory state may upregulate TNF-α in the body, making anti-TNF treatment particularly effective (28, 29). Although the risk of infection must be monitored, under close observation, adalimumab is relatively safe for those with renal insufficiency and has minimal impact on blood glucose levels, which is one of its advantages over traditional immunosuppressants.

Multiple studies indicate that ADA treatment is an effective strategy for treating refractory VKH patients. Namba (30) and Khan (31) evaluated the safety and efficacy of ADA in non-infectious uveitis, confirming its good safety and effectiveness; Takeuchi M. conducted a multicenter clinical trial, finding that ADA treatment could significantly improve the central fovea choroidal thickness (SFCT), IGCA examination scores, and reduce corticosteroid dosage, with significant visual improvement in patients with sunset glow fundus (24). Multicenter studies on adalimumab have indicated good safety and efficacy, with most patients in good systemic condition. The uniqueness of this case lies in the coexistence of multiple severe comorbidities (diabetes, late-stage CKD), which pose greater challenges and requirements for the safety of ADA treatment. Nonetheless, under the premise of closely monitoring liver and kidney function and blood glucose levels, ADA still demonstrates good tolerance and significant anti-inflammatory effects, effectively controlling ocular inflammation and preventing further deterioration of vision.

## Conclusion

The management of corticosteroid therapy in VKH patients with concomitant DR warrants careful consideration. Although the coexistence of VKH, diabetic retinopathy, and chronic kidney disease is uncommon, for patients with both diseases, monitoring blood glucose levels during treatment and the rational use of corticosteroids, immunosuppressants, and biologics are crucial.

## Data Availability

The original contributions presented in the study are included in the article/supplementary material, further inquiries can be directed to the corresponding author.
